# Floridoside Exhibits Antioxidant Properties by Activating HO-1 Expression via p38/ERK MAPK Pathway

**DOI:** 10.3390/md18020105

**Published:** 2020-02-10

**Authors:** Tingting Niu, Gaoqing Fu, Jiawei Zhou, Hui Han, Juanjuan Chen, Wei Wu, Haimin Chen

**Affiliations:** State Key Laboratory for Managing Biotic and Chemical Threats to the Quality and Safety of Agro-products, Ningbo University, Ningbo 315211, China

**Keywords:** floridoside, hepatocyte, antioxidant, Nrf2/HO-1 pathway

## Abstract

Floridoside is a low-molecular-weight organic compound, which can be accumulated by red algae under stressful conditions to protect cells via its excellent antioxidant properties. In the present study, we investigated the antioxidant mechanism of floridoside toward human hepatocyte L-02 cells. We found that floridoside had no toxicity to L-02 cells, and no reactive oxidative species were induced by it either. However, the expression of hemoxygenase-1 (HO-1) protein was up-regulated upon exposure to floridoside, and two antioxidant enzymes, superoxide dismutase (SOD) and GSH-Px, were activated by floridoside. Moreover, we investigated the pathway involved in the production of these antioxidants, p38/extracellular signal-regulated kinase (ERK) MAPK-nuclear factor-erythroid-2-related factor 2 (Nrf2) pathway. ERK1/2 and p38 phosphorylation, nuclear translocation of Nrf2, and activation of ARE luciferase activity were observed upon exposure to floridoside. siRNA interference and inhibitor treatment suppressed the HO-1 expression and the phosphorylation of ERK1/2 and p38, respectively. These results indicated that floridoside exerted its antioxidant activity by activating HO-1 expression via p38/ERK MAPK-Nrf2 pathway in human hepatocyte L-02 cells.

## 1. Introduction

Floridoside, a 2-*O*-d-glycerol-α-d-galactoside, has long been known as a typical low-molecular-weight red algal carbohydrate. It is not only the main photosynthetic product of many *Rhodophyceae*, but also plays a role in cell protection under many stressful conditions, such as hyperosmolality or high temperature [1,2]. The antioxidant activity of floridoside helps red algae fight, in particular, the reactive oxygen species (ROS) in these stressful environments. Ochsenkühn et al. have indicated that floridoside has an ability to prevent salinity- and heat-stress-induced ROS production [3]. Therefore, researchers are also curious about its biological activity in humans. Since Colin and Gueguen have isolated floridoside in 1930, the biological activities of floridoside have been widely studied, such as immune regulatory [4], anti-inflammatory [5], anti-bacterial [6], and antioxidant properties [7]. Li et al. have reported that floridoside isolated from *Laurencia undulata* inhibits ROS production and improves expression of glutathione and superoxide dismutase (SOD) genes [8]. However, the molecular mechanism underlying its antioxidant activity remains largely unexplored.

Liver is the main organ for metabolizing endogenous and exogenous substances. Alcohol, chemicals, and infections can cause liver damage [9,10]. Studies have shown that diseases, such as cirrhosis, liver cancer, and hepatitis, are thought to be induced by oxidative stress [11,12,13]. It can not only cause liver cell injury and death by changing intracellular macromolecules (including DNA, proteins, and lipids), but also regulate the death of liver cells by altering signal transduction pathway [14,15]. Therefore, it is urgently necessary to develop effective hepatoprotective agents for the prevention and treatment of liver diseases. Due to the particularity of liver medication, hepatoprotective drugs should be safe and non-toxic to humans. In recent years, a great deal of attention has been paid to the application of small-molecule polyhydroxy compounds in liver diseases. Ren et al. have reported that quercetin nanoparticles exhibit antitumor activity by inhibiting the proliferation and inducing the apoptosis of liver cancer cells [16]. Hsiang et al. have found that silymarin regulates the expressions of genes relevant to apoptosis and oxidative stress in HepG2 cells via the suppression of NF-κB activity [17]. Furthermore, our early study has indicated that floridoside can promote the growth of hepatocytes L-02 cells and protect cells from the reduction of mitochondrial membrane and apoptosis caused by H_2_O_2_ through free-radical scavenging [18]. However, the antioxidant mechanism of floridoside has not been fully illuminated in hepatocytes.

Nuclear factor-erythroid-2-related factor 2 (Nrf2)/antioxidant response element (ARE) antioxidant pathway is the most important endogenous antioxidant signaling pathway discovered so far [19,20]. Many antioxidants, such as flavonoids, polyphenols, and carotenoids, can exert antioxidant effects by activating this pathway [21,22,23]. However, it still remains unknown whether floridoside can also activate such pathway.

**Figure 1:**
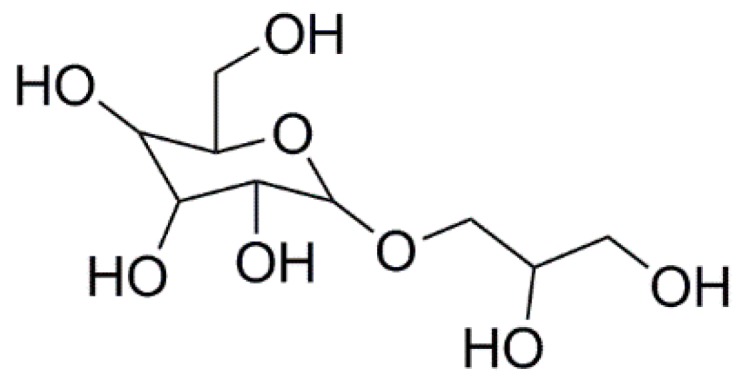
The structure of floridoside isolated from *P. haitanensis.*

In the present study, we aimed to investigate the antioxidant mechanism of floridoside isolated from *Pyropia haitanensis* (Figure 1) in L-02 cells and clarify whether the Nrf2/ARE signaling pathway was also involved in the antioxidant effect of floridoside.

## 2. Results

### 2.1. Effect of Floridoside on Viability of L-02 Cells

**Figure 2:**
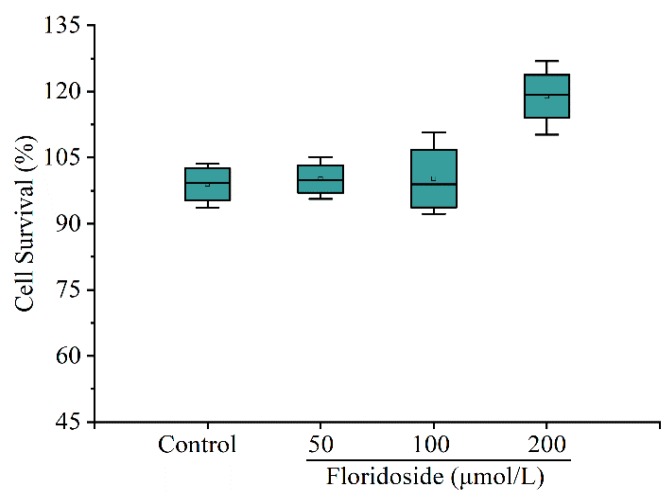
Effect of floridoside on L-02 cell survival. Cells were exposed to 50, 100, and 200 μmol/L floridoside for 2 h. Data were expressed as the mean ± SD (*n* = 6).

Figure 2 shows that floridoside exposure had no cytotoxic effects, and the cell survival was even increased upon exposure to 200 μmol/L floridoside. Compared with controls, the cell survival was increased by 18.92% after exposure to 200 μmol/L floridoside for 2 h.

### 2.2. Effect of Floridoside on Intracellular ROS Production

**Figure 3:**
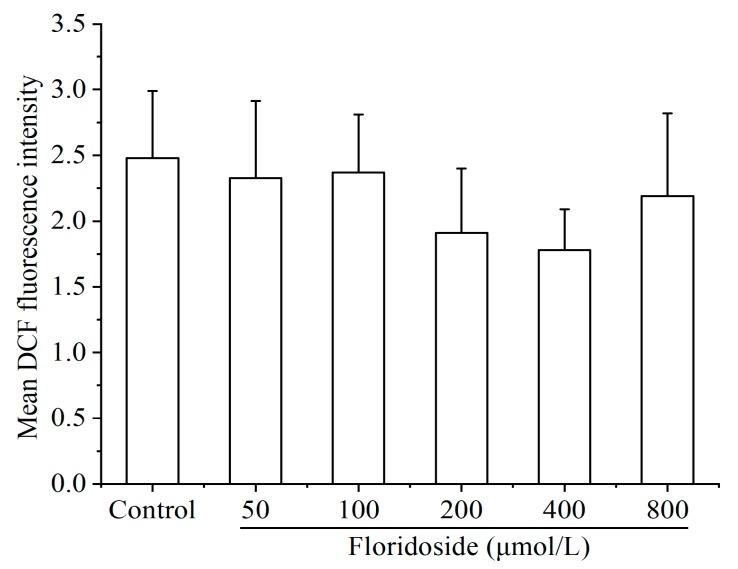
Effect of floridoside on intracellular reactive oxygen species (ROS) production in L-02 cells. L-02 cells were incubated with 50, 100, 200, 400, and 800 μmol/L floridoside for 2 h. Intracellular ROS were detected by commercial DCFH-DA probes.

Different concentrations of floridoside (50–800 μmol/L) failed to increase the intracellular ROS level. In contrast, decreased ROS production was observed in cells, although there was no significant difference. Compared with controls, the intracellular ROS level was reduced by up to 24% upon exposure to different concentrations of floridoside (Figure 3).

### 2.3. Effect of Floridoside on Enzyme Activities of SOD and GSH-Px in L-02 Cells

**Figure 4:**
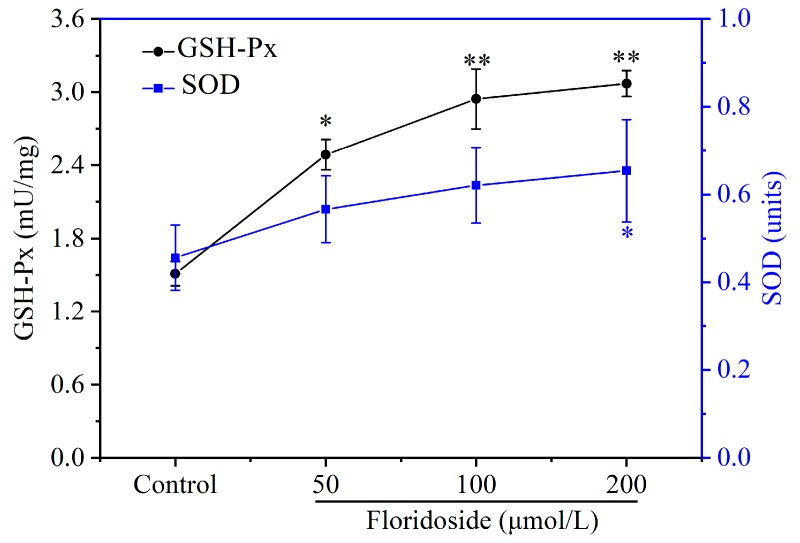
Effect of floridoside on superoxide dismutase (SOD) and GSH-Px enzyme activity. Cells were exposed to 50, 100, and 200 μmol/L floridoside for 2 h. Data were expressed as the mean ± SD. * *p* < 0.05 and ** *p* < 0.01, compared with controls.

The effect of floridoside on antioxidant activities (SOD and GSH-Px) was assessed. Figure 4 reveals that both SOD and GSH-Px enzyme activities were increased after exposure to different concentrations of floridoside. However, the elevation of SOD activity induced by 50 and 100 μmol/L floridoside was not significant, while 200 μmol/L floridoside significantly increased the SOD activity (*p* < 0.05). Different concentrations of floridoside obviously increased the GSH-Px activity (*p* < 0.05), especially the exposure at 200 μmol/L, which showed a 2.02-fold increase compared with controls.

### 2.4. Effect of Floridoside on HO-1 Expression in L-02 Cells

**Figure 5:**
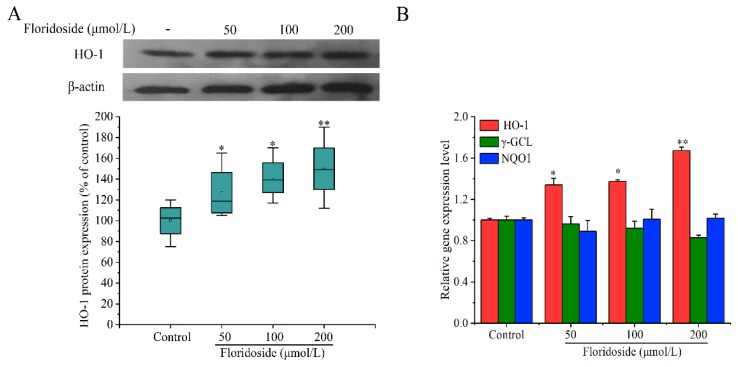
Effect of floridoside on protein expression of HO-1 and mRNA expression of *HO-1*, *γ*-glutamyl cysteine ligase (*γ-GCL*), and NAD(P)H: quinine oxidoreductase 1 (*NQO1*) in L-02 cells. (**A**) HO-1 protein expression was examined by western blotting analysis. (**B**) Expressions of *HO-1*, *γ-GCL*, and *NQO1* at the mRNA level were examined by RT-qPCR. Cells were exposed to 50, 100, and 200 μmol/L floridoside for 2 h. * *p* < 0.05 and ** *p* < 0.01, compared with controls.

HO-1 protein plays a vital role in cell antioxidant activity. In the present study, we assessed whether floridoside could up-regulate the HO-1 expression at the protein level by western blotting analysis. Figure 5A shows that the expression of HO-1 was significantly increased after exposure to floridoside for 2 h (*p* < 0.05). Compared with controls, the HO-1 expression was increased by 27.7%, 41.3%, and 49.9% after exposure to three different concentrations of floridoside, respectively.

Next, the expressions of *HO-1*, *γ*-glutamyl cysteine ligase (*γ-GCL*), and NAD(P)H: quinine oxidoreductase 1 (*NQO1*) at the mRNA level were examined by RT-qRCR. Figure 5B indicates that exposure to 200 μmol/L floridoside significantly up-regulated the expression of *HO-1* at the mRNA level, showing a 1.67-fold increase compared with controls, which was consistent with the western blotting analysis. However, floridoside exposure did not increase the expressions of *γ-GCL* and *NQO1* at the mRNA level compared with controls.

### 2.5. Effect of Floridoside on Nrf2/ARE Pathway in L-02 Cells

**Figure 6:**
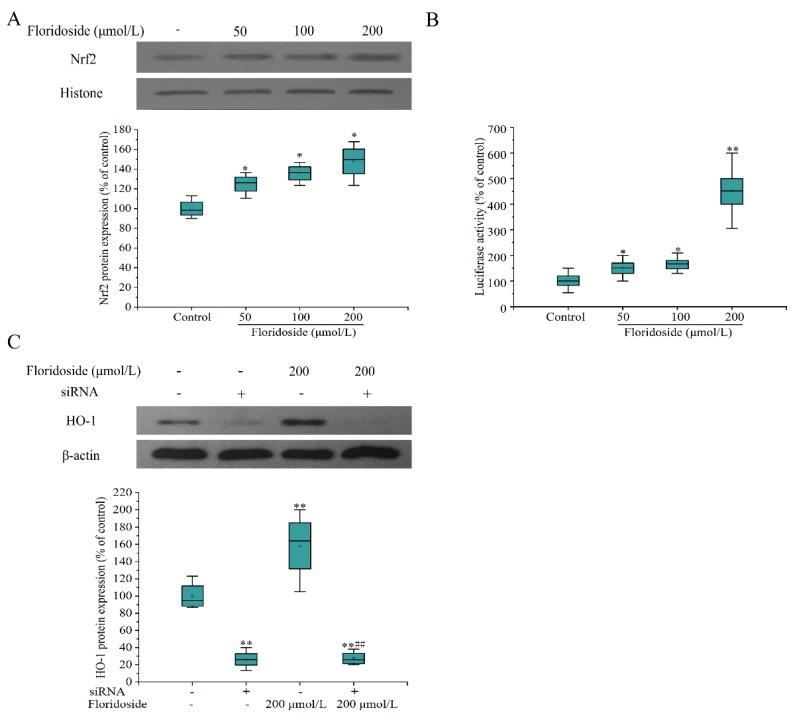
Effect of floridoside on nuclear accumulation of nuclear factor-erythroid-2-related factor 2 (Nrf2), ARE luciferase activity, and HO-1 protein expression in L-02 cells. (**A**) L-02 cells were exposed to 50, 100, and 200 μmol/L floridoside for 2 h. The Nrf2 nuclear proteins were examined by western blotting analysis. (**B**) L-02 cells were transiently transfected with p-ARE-Luc reporter plasmid for 24 h and then exposed to 50, 100, and 200 μmol/L floridoside for 2 h. (**C**) Cells were transiently transfected with *Nrf2* siRNA for 8 h and then exposed to 200 μmol/L floridoside for 48 h. HO-1 protein expression was determined by western blotting analysis. * *p* < 0.05 and ** *p* < 0.01, compared with controls; ## *p* < 0.01, compared with floridoside-alone treatment group.

We next examined whether floridoside treatment could activate Nrf2/ARE pathway. Figure 6A shows that the expression of nuclear Nrf2 at the protein level was significantly increased compared with controls after exposure to various concentrations of floridoside for 2 h (*p* < 0.05). Furthermore, the ARE luciferase activity was examined (Figure 6B). The ARE luciferase activity was significantly increased with the increase of floridoside concentration, showing a 4.5-fold increase after exposure to 200 μmol/L floridoside. Finally, *Nrf2* siRNA interference was used to detect the effect of *Nrf2* interference on downstream regulation. The interference efficiency of siRNA was about 74%. After siRNA interference, floridoside exposure failed to restore the expression of HO-1 protein to the normal level (Figure 6C).

### 2.6. Effect of Floridoside on MAPK Pathway

**Figure 7:**
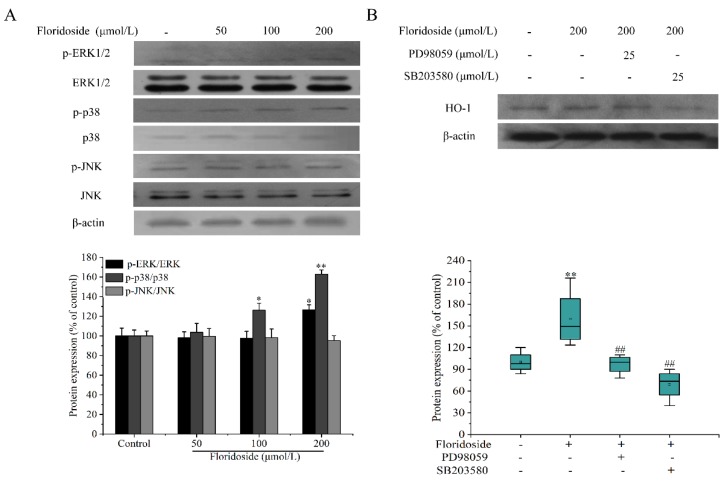
Effect of floridoside on MAPK pathway and HO-1 protein expression in L-02 cells. (**A**) Cells were treated with 50, 100, and 200 μmol/L floridoside for 2 h. Whole cells lysates were prepared and analyzed by western blot. (**B**) L-02 cells were pretreated with 25 μmol/L PD98059 or SB203580 for 1 h, and then stimulated with 200 μmol/L floridoside for 2 h. HO-1 protein expression was determined by western blot. * *p* < 0.05 and ** *p* < 0.01, compared with control; ## *p* < 0.01, compared with floridoside-alone treatment group.

The effect of floridoside on mitogen-activated protein kinases (MAPK) pathway was also examined. Figure 7A indicates that high concentrations (100 and 200 μmol/L) of floridoside significantly increased the expression of p-p38 at the protein level (*p* < 0.01). Compared with controls, the expression of p-p38 at the protein level was increased by 26.3% and 62.8% after exposure to high concentrations of floridoside. Moreover, we found that 50 and 100 μmol/L floridoside could not increase the phosphorylation of extracellular signal-regulated kinase (p-ERK), while only 200 μmol/L floridoside markedly increased the expression of p-ERK at the protein level. Floridoside treatment had no effect on the expression of p-JNK at the protein level. Furthermore, we explored whether the expressions of p38 and ERK at the protein level were associated with Nrf2/ARE pathway. We used specificity inhibitors of SB203580 (p38) and PD98059 (ERK) to block the expressions of p38 and ERK. We found that PD98059 inhibited the expression of HO-1 protein, whereas SB203580 not only suppressed the HO-1 protein expression induced by flosidoside, but also decreased the endogenic HO-1 protein expression. Compared with controls, the expression of HO-1 at the protein level was decreased by 22% (Figure 7B).

## 3. Discussion

Floridoside is a small-molecule carbohydrate synthesized by red algae during photosynthesis. It has a wide range of applications, such as skin care additive [24], protective agent in ultra-low temperature refrigeration [25,26], and fish food additive [27]. In our previous study, we have found that floridoside can restore the decreased antioxidant enzyme activity and mitochondrial membrane potential caused by H_2_O_2_ challenge [18]. The results in this study showed that high concentration of floridoside alone promoted the proliferation of L-02 cells in 2 h and significantly increased the antioxidant activities of GSH-Px and SOD. However, Li et al. found that cells (MRC-5, Raw264.7, HL-60, and HT-1080) that were treated with 1, 10, 25, and 100 μmol/L floridoside for 24 h cannot promote the cell proliferation [8]. Kim et al. also reported that floridoside (1, 10, and 50 μmol/L) treatment for 24 h had no effect on BV2 cell proliferation [5]. Therefore, the reason for promoting rapid cell proliferation of floridoside requires further investigation.

In addition, some antioxidants have two side effects. For example, Vc and VE have strong antioxidant activity at low concentrations, but may be toxic to cells at high concentrations [28,29]. To investigate whether floridoside has such characteristic, we examined the effect of floridoside (50 to 800 μmol/L) on intracellular ROS level, and the results showed that floridoside failed to increase the ROS production and suggested that floridoside is safe and has no prooxidative properties within the concentration ranging from 50 to 800 μmol/L. Therefore, we further investigated the antioxidant mechanism of floridoside in L-02 cells.

Cellular defensive properties of small-molecule polyhydroxy compounds, such as resveratrol and epigallocatechin-3-gallate (EGCG), can be explained through direct and indirect ways. They can not only directly scavenge free radicals and quench singlet oxygen, but also indirectly activate endogenous Nrf2/ARE signaling pathways. Truong et al. have reviewed the two ways of resveratrol in regulating cellular defense systems against oxidative stress [30]. Kanlaya et al. found that EGCG protects renal tubular cells from oxalate-induced epithelial mesenchymal transition via Nrf2 pathway [31]. Floridoside also belongs to small-molecule polyhydroxy compounds. Early studies have shown that the antioxidant mechanism of floridoside is mainly through free-radical scavenging [8]. It remains unknown whether floridoside also has a potential to activate Nrf2/ARE pathway. The results in this study showed that floridoside exposure caused the nuclear translocation of Nrf2, increased the ARE luciferase activity, and up-regulated the *HO-1* expression at the mRNA level, indicating that floridoside might activate the Nrf2/ARE signaling pathway. Subsequently, siRNA interference dramatically decreased the HO-1 protein expression, and floridoside exposure had no effect on such reduction, indicating that floridoside indeed had an ability to activate Nrf2/ARE pathway.

**Figure 8:**
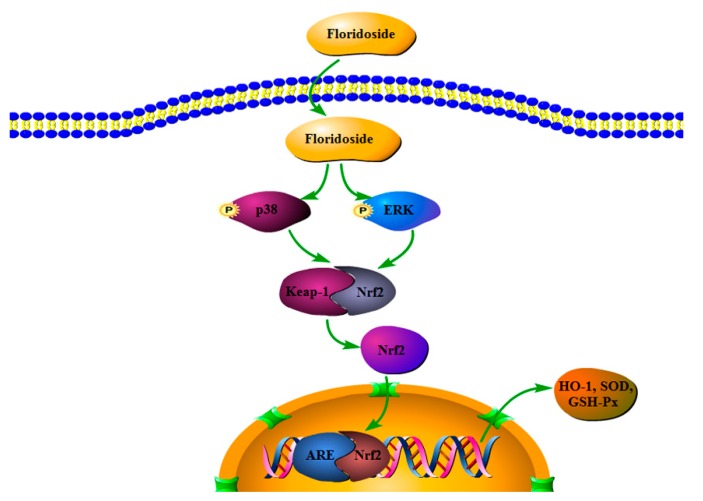
Antioxidant mechanism of floridoside in L-02 cells.

It has been reported that Nrf2 is the substrate of several protein kinases, including protein kinase C (PKC), phosphatidylinositol 3-kinase (PI3K), ERK, and p38 [32,33,34]. We examined the effect of floridoside on MAPK pathway of Nrf2 upstream signaling pathway. The results showed that floridoside activated the MAPK pathway through phosphorylation of p38 and ERK (200 μmol/L). Furthermore, we found that inhibitors (SB203580 and PD98059) blocked the expression of HO-1 protein, and floridoside failed to increase the expression of HO-1 protein. Therefore, we speculated that floridoside activated Nrf2-mediated HO-1 protein expression through p38 and ERK MAPK pathways in L-02 cells (Figure 8).

## 4. Materials and Methods

### 4.1. Cell Culture

Human hepatocyte line L-02 was purchased from China Center for Type Culture Collection (CCTCC, Wuhan, China) and maintained in Dulbecco’s Modified Eagle’s medium (DMEM) supplemented with 10% (V/V) fetal bovine serum (FBS) (Gibco, Thermo Fisher Scientific, Inc., Waltham, MA, USA) at 37 °C in a humidified atmosphere containing 5% CO_2_. Cells were cultured to 70%–80% confluence and then exposed to different concentrations of floridoside isolated from *P. haitanensis* (Xiangshan, Ningbo, China) (purity ≥92.3%) for 2 h.

### 4.2. Cell Viability Assay

L-02 cells were seeded into 96-well plates and exposed to 50, 100, and 200 μmol/L floridoside for 2 h, followed by incubation with 20 μL 3-[4,5-dimethylthiazol-2-yl]-2,5-diphenyltetrazolium bromide (MTT) at 37 °C for 4 h. Subsequently, culture medium was removed, and 150 μL dimethyl sulfoxide was added. Spectrophotometric absorbance of the samples was determined at a wavelength of 492 nm, and the data were normalized to the control group.

### 4.3. ROS Detection

Commercial DCFH-DA probes were used to detect the effects of floridoside on intracellular ROS. Cells were exposed to 50, 100, 200, 400, and 800 μmol/L floridoside for 2 h, followed by incubation with FBS-free medium supplemented with 20 μmol/L DCFH-DA probe at 37 °C for 45 min. Beckman Gallios Flow Cytometer (Beckman Counter, Inc., Brea, CA, USA) was used to determine the intracellular ROS level, and the data were expressed as mean dichlorofluorescein (DCF) fluorescence intensity.

### 4.4. Antioxidant Activity Assay

L-02 cells were cultured in 6-well plate in the presence of 50, 100, and 200 μmol/L floridoside for 2 h. Subsequently, cells were washed twice with ice-cold PBS and lysed in lysis buffer (Beyotime, Shanghai, China) for 30 min on ice. The cell lysates were centrifuged at 12,000 rpm for 10 min at 4 °C. Enzyme activities of SOD and glutathione peroxidase (GSH-Px) were determined using commercial enzyme activity assay kits (Beyotime, Shanghai, China) according to manufacturer’s instructions.

### 4.5. Western Blotting Analysis

L-02 cells was exposed to different concentrations of floridoside for 2 h. In parallel experiments, L-02 cells were pretreated with 25 μmol/L PD98059 (S1805, Beyotime, Shanghai, China) or SB203580 (S1863, Beyotime, Shanghai, China) for 1 h, and then stimulated with 200 μmol/L floridoside for 2 h. Nuclear extracts were prepared using the Nuclear and Cytoplasmic Protein Extration kit (P0027, Beyotime, Shanghai, China) according to manufacturer’s instructions. Total proteins were lysed and extracted according to manufacturer’s instructions (P1103, Beyotime, Shanghai, China), and protein concentration was determined using Easy II protein quantitative kit (BCA) (TransGen, Beijing, China). For western blotting analysis, equal amounts of proteins (30 μg) in the nuclear extracts or in the whole cell lysates were subjected to 10% sodium dodecylsulfate-polyacrylamide gel (SDS-PAGE) and then electro-transferred onto polyvinylidene fluoride (PVDF) membranes. The membranes were blocked with 5% skim milk at room temperature for 2 h, washed with Tris-buffered saline containing Tween-20 (TBST) for 3 × 8 min, and incubated with antibodies against hemoxygenase-1 (HO-1) (1:2000), phospho-ERK1/2 (1:1000), phospho-p38 (1:1000), and phospho-JNK (1:1000) (Cell Signaling Technology, USA); Nrf2 (1:1000), ERK (1:1000), JNK (1:1000), p38 (1:1000), and β-actin (1:1000) (Santa Cruz Biotechnology, CA, USA) overnight at 4 °C. Subsequently, blots were incubated with appropriate horseradish-peroxidase (HRP)-conjugated secondary antibodies (mouse anti-rabbit IgG, 1:2000, goat anti-mouse IgG, 1:8000, (Santa Cruz Biotechnology, CA, USA)) at room temperature for 1 h, and then washed with TBST for 3 × 8 min. The immunoreactive bands were visualized by WesternBright ECL-HRP Substrate (Advansta Inc., Menlo Park, CA, USA). The band intensity was quantified by using ImageJ software, and β-actin was used as a loading control.

### 4.6. Real-Time Quantitative PCR (RT-qPCR)

After floridoside challenge, cells were harvested, and total RNA was isolated with RNA-Solv^®^ Reagent (Omega Bio-tek Inc., Norcross, GA, USA) according to the manufacturer’s Instructions. Briefly, 1 μg of total RNA was reversely transcribed into first-strand cDNA in a 20-μL reverse transcription (RT) reaction system, and RT-qPCR was conducted with 1 μL of RT product on a LightCycler 96 Real-Time PCR System (Roche, Switzerland) using SYER-Green I. Four pairs of specific primers were designed as follows [35]: *HO-1*-F: AAGTATCCTTGTTGACACG and *HO-1*-R: TGAGCCAGGAACAGAGTG; *NQO1*-F: AGACCTTGTGATATTCCAGTTC and *NQO1*-R: GGCAGCGTAAGTGTAAGC; *γ-GCL*-F: CAGTGGTGGATGGTTGTG and *γ-GCL*-R: ATTGATGATGGTGTCTATGC; *β-actin*-F: CGGTGAAGGTGACAGCAG and *β-actin*-R: TGTGTGGACTTGGGAGAGG. The relative expression levels of target genes were calculated with the 2^-ΔΔCt^ method, and *β-actin* was selected as a housekeeping gene for RT-qPCR.

### 4.7. Transient Transfection and Luciferase Reporter Assays

L-02 cells were transiently co-transfected with 1 μg of firefly luciferase reporter plasmid p-ARE-Luc (Beyotimes, Shanghai, China) and 0.1 μg of p-RL. Transfection was performed using X-tremeGENE HP DNA Transfection Reagent (Roche, Switzerland) according to the manufacturer’s instructions. After transfection for 24 h, cells were exposed to floridoside for 2 h. Firefly and Renilla luciferase activities were determined in cell lysates using the Dual-Glo^®^ Luciferase Assay System (Promega Corp., Madison, WI, USA). All experiments were performed in triplicate, and the luciferase activity was normalized using Renilla luciferase activity.

### 4.8. Nrf2 siRNA Interference Assay

The siRNA duplexes were synthesized by GenePharma (Shanghai, China). *Nrf2* siRNA duplex with the following sense and antisense was used, 5′-GGAGGCAAGAUAUAGAUCUTT-3′ (sense) and 5′-AGAUCUAUAUCUUGCCUCCTT-3′ (antisense). L-02 cells were seeded into 6-well plates until 70%–80% confluence, and the medium was replaced with OPTI-MEM reduced serum medium. Transient transfection of siRNAs was conducted using X-tremeGENE siRNA Transfection Reagent (Roche, Switzerland). siRNA (2 μg) and X-tremeGENE siRNA Transfection Reagent (10 μL) were briefly diluted into 100 μL OPTI-MEM reduced serum medium, followed by incubation at room temperature for 5 min. Diluted X-tremeGENE siRNA Transfection Reagent was added into siRNA dilution, followed by incubation at room temperature for 20 min, and the mixed transfection compound was directly added to the cells. After transfection for 8 h, the medium was replaced, and the cells were exposed to 200 μmol/L floridoside for 48 h. The transfection efficiency was determined by western blotting analysis.

### 4.9. Statistical Analysis

The results were expressed as mean ± SD, and statistical analyses were performed using the SPSS software, version 16.0 (SPSS Inc., Chicago, IL, USA). The statistical significance was analyzed by one-way ANOVA. *P* < 0.05 was regarded as statistically significant.

## 5. Conclusions

Collectively, we, for the first time, reported the antioxidant mechanism of floridoside in hepatocytes. The antioxidant mechanism of floridoside was activated by Nrf2-mediated HO-1 protein expression through ERK1/2 and p38 MAPK pathways. Furthermore, floridoside was safe and could not induce ROS production at high concentrations (up to 800 μmol/L) as isoeugenol, quercetin, and morin. Taken together, our findings provided valuable insights into the development of floridoside products and its application in the biomedical field.
